# Aflatoxin B1 Tolerance and Accumulation in Black Soldier Fly Larvae (*Hermetia illucens*) and Yellow Mealworms (*Tenebrio molitor*)

**DOI:** 10.3390/toxins9060185

**Published:** 2017-06-02

**Authors:** Guido Bosch, H. J. van der Fels-Klerx, Theo C. de Rijk, Dennis G. A. B. Oonincx

**Affiliations:** 1 Animal Nutrition Group, Department of Animal Sciences, Wageningen University & Research, P.O. Box 338, 6700 AH Wageningen, The Netherland; 2 RIKILT, Wageningen University & Research, P.O. Box 230, 6700 AE Wageningen, The Netherland; theo.derijk@wur.nl; 3 Laboratory of Entomology, Department of Plant Sciences, Wageningen University & Research, P.O. Box 16, 6700 AA Wageningen, The Netherland; dennis.oonincx@wur.nl

**Keywords:** aflatoxins, *Hermetia illucens*, food waste mitigation, livestock feed, novel protein source, *Tenebrio molitor*

## Abstract

Crops contaminated with fungal mycotoxins such as aflatoxin B1 (AFB1) are often downgraded or removed from the food chain. This study aimed to evaluate the tolerance and accumulation of AFB1 in two insect species to determine whether they could be used to retain condemned mycotoxin contaminated crops in the food chain. First, instar black soldier fly larvae (*Hermetia illucens*, BSF) and yellow mealworm (*Tenebrio molitor*, YMW) were fed poultry feed spiked with AFB1 and formulated to contain levels of 0.01, 0.025, 0.05, 0.10, 0.25, and up to 0.5 mg/kg dry feed. Poultry feed without any additions and feed with only the solvent added served as controls. The AFB1 in the feed did not affect survival and body weight in the BSF and YMW larvae (*p* > 0.10), indicating a high tolerance to aflatoxin B1 in both species. Furthermore, AFB1 and aflatoxin M1 (AFM1) were below the detection limit (0.10 µg/kg) in BSF larvae, whereas the YMW had AFB1 levels that were approximately 10% of the European Union’s legal limit for feed materials and excreted AFM1. It is concluded that both BSF larvae and YMW have a high AFB1 tolerance and do not accumulate AFB1.

## 1. Introduction

The combination of the human population growing to 9 billion in 2050, increasing living standards, and urbanisation in developing countries fuels the global demand for food and in particular animal-derived protein [1]. By 2050, the global food demand is estimated to be increased by 70% and that of meat by 85%, relative to the period from 2005 to 2007 [2]. Increasing food production is highly challenging because required resources such as land, water, and fossil energy are limited, and the environmental impact of crop and livestock production already needs to be minimised [3,4]. Next to increasing food production, reducing waste in the food supply chain will contribute to global food security [1,4]. Within the food supply chain, suboptimal conditions on the field and during storage may lead to the growth of endophytic and saprophytic fungi that can produce mycotoxins [5]. Mycotoxin-contaminated cereals (e.g., maize, wheat, barley) and nuts (e.g., peanut, walnuts) can be deleterious to humans [6] and can reduce animal health and production [5,7,8]. Countries around the world, therefore, tighten their regulations and define maximum allowable mycotoxin concentrations in food and feed products. Furthermore, mycotoxin presence in these products is monitored and, when concentrations are above maximum allowable limits, products are downgraded or removed from the food chain [9].

One of the major agriculturally important mycotoxins are aflatoxins produced by the *Aspergillus* genus. Of the four aflatoxins, aflatoxin B1 (AFB1) is the most toxic and occurs most frequently [5]. Maximum allowable concentrations for the presence of AFB1 have therefore been defined in countries around the world, e.g., 0.02 mg/kg for feed material relative to feed materials containing 12% moisture in the European Union [10]. Several factors contributing to the presence of mycotoxins in crops are beyond human control [5]. Therefore, it is of interest to find strategies that can reduce mycotoxin presence so condemned crops can be retained in the food supply chain.

Insects can be used as food by both humans and animals and as such have been proposed to contribute to food security [11]. Some insect species thrive under challenging conditions by nature. Exposure to fungi and their products during insect evolution have resulted in coping capabilities allowing insects to take advantage of mycotoxin-contaminated but nutritional food sources. Wild-type laboratory strains of the common fruit fly (*Drosophila melanogaster*), which feed on yeast growing on fruit [12], showed variable resistance (i.e., egg-to-adult viability) to AFB1 in their medium up to 0.4 mg/L [13] and to AFB1, aflatoxin B2, aflatoxin G1 and sterigmatocystin up to 4 mg/L [14]. Variation in resistance to AFB1 was also found between natural fruit fly populations from different areas, and was correlated with the aflatoxin presence in their area of origin [15]. Furthermore, profound differences in AFB1 tolerance were found between larvae of the navel orangeworm (*Amyelois transitella*), that specifically feed on unharvested fruits, and the corn earworm (*Helicoverpa zea*), that only occasionally encounters aflatoxin-releasing fungi [16]. Dietary AFB1 levels up to 20 mg/kg fed to first instar larvae of the navel orangeworm resulted in a normal pupation rate and pupal weight [16], whereas a dietary level of 0.2 mg/kg resulted in a mortality of 55% after 9 days and 100% after 15 days in first instars of the corn earworm [17]. Tolerance for AFB1 may increase with age, as observed in cabbage looper (*Trichoplusia ni*), with no effects on pupal weight and pupation rate at 0.02 mg/kg AFB1 for first instars, 0.2 mg/kg for 5-day old, 1 mg/kg for 7-day old, and 3 mg/kg for 10-day old larvae [18].

While these insect species are not considered as future proteinaceous food or feed sources, there is growing interest in larvae of the black soldier fly (*Hermetia illucens*, BSF) and the yellow mealworm (*Tenebrio molitor*, YMW). These species have the capacity to efficiently convert various organic waste into body mass [19,20]. As such, YMW and BSF larvae can be sustainable proteinaceous feed ingredients for pigs, poultry, and fish, as well as alternatives for conventional sources (soybean meal and fishmeal) with associated environmental and societal issues [21]. The mycotoxin tolerance of these insect species is unknown. In nature, the BSF larvae thrive in various decomposing materials including plants, animals, and manure [22]. Furthermore, the larvae are commonly reared on diets mixed with water to approximately 70% moisture and kept at 28 °C [23,24], conditions that promote bacterial and fungal growth. The saprophagous BSF larvae are commonly exposed to fungi and their products, which might have resulted in coping capabilities and tolerance to mycotoxins as found in the aforementioned insect species. Yellow mealworms are a well-known pest of stored grain products. When in the dark, this species aggregates in moist areas which are likely to contain fungi [25]. Depending on the species of mycotoxin-producing *Fusarium*, YMW avoided (*F*. *avenaceum*, *F*. *bassiana*) or preferred (*F*. *proliferatum*, *F*. *poae*, *F*. *culmorum*) to feed on infected kernels [26]. Few data are available on the impact of specific mycotoxins in the diet on YMW. Growth was reduced but mortality was not affected in YMW fed a diet containing 450 mg/kg fumonisin B1 [27] or 128 mg/kg T2-toxin [28], two mycotoxins produced by *Fusarium* fungi. Furthermore, it was estimated that 39% of the fumonisin B1 intake was excreted [27]. Data on the possible accumulation or excretion of AFB1 in BSF and YMW are lacking. The aim of the current study was therefore to investigate the tolerance and accumulation of AFB1 by larvae of the BSF and the YMW.

## 2. Results

### 2.1. Experimental Feeds

The analysed AFB1 concentrations of the spiked poultry feed were 82 to 98% of the formulated contents, except for the feed formulated to contain 0.01 mg/kg, which had a higher analysed value (Table 1). The coefficients of variation for the analysed AFB1 content in the 0.25 and 0.5 mg/kg feeds were respectively 3.0 and 5.6%, indicating a homogenous sample material and repeatable analysis.

### 2.2. Insect Rearing and Impact of Solvent

Feeding the poultry feed without the solvent resulted in high survival rates for both species (Table 2), which indicates that the applied rearing conditions were suitable to support the development of both insect species. The solvent used as a vehicle to spike the poultry feed with AFB1 contained methanol and chloroform, which both are potentially toxic. No differences in body weight and survival were found between the BSF larvae fed the solvent-containing poultry feed or the feed without the solvent (*p* > 0.10). For the YMW, survival was similar between dietary treatment groups (*p* > 0.10), but larvae fed the solvent-containing poultry feed tended to have a lower body weight than the larvae fed the feed without the solvent (*p* = 0.088). As methanol and chloroform are both highly volatile at ambient temperatures, the solvent likely evaporated during the thorough mixing of the feed, rendering it free from these compounds.

### 2.3. Aflatoxin Tolerance and Accumulation

**Figure 1:**
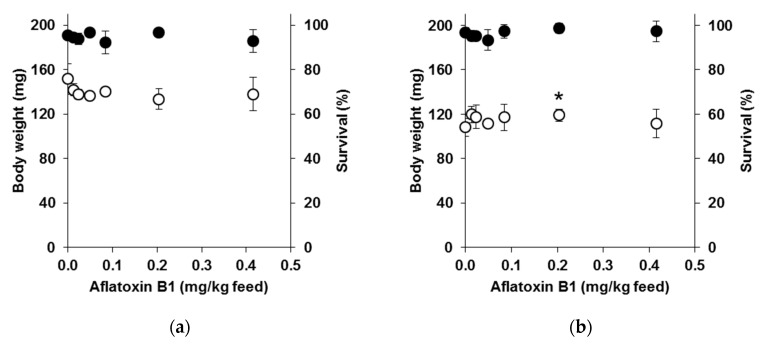
(**a**) Mean body weight (○) and survival (●) (±SD, *n* = 3 replicates) of black soldier fly larvae provided with poultry feed spiked with varying levels of aflatoxin B1 for 10 days; (**b**) Mean body weight (○) and survival (●) (±SD, *n* = 3 replicates) of yellow mealworms provided with poultry feed spiked with varying levels of aflatoxin B1 until first pupae emergence (approximately 40 days). Yellow mealworms fed 0.204 mg/kg aflatoxin B1 had a higher body weight (*, *p* < 0.05) than those provided with the feed without aflatoxin B1.

Both insect species tolerated AFB1 in their feed up to the highest level of 0.415 mg/kg. Similar body weights and survival rates were found for the BSF larvae fed the poultry feed containing varying levels of AFB1 compared to those fed the feed with only the solvent (*p* > 0.10) (Figure 1a). Similar findings were obtained for the YMW, except for the larvae fed the feed containing 0.204 mg/kg AFB1, which were heavier than those fed the feed without AFB1 (*p* = 0.035) (Figure 1b). The high survival rates and normal growth indicate that the AFB1 tolerance was present in all instars of the BSF and YMW larvae.

The AFB1 and aflatoxin M1 (AFM1) concentrations in the BSF larvae were below the detection limit (i.e., 0.10 µg/kg) (Table 3). The YMW larvae had low AFB1 concentrations when provided 0.023 mg AFB1/kg feed and AFM1 concentrations were below the detection limit. These findings suggest that the larvae rapidly excreted or catabolised the AFB1 after ingestion. However, part of AFB1 might also have been bound to proteins [29] and left undetected.

The mass balance indicates that considerable amounts of AFB1 were lost for both insect species. For the BSF larvae, the amount of AFB1 lost varied from 83.0 to 95.1% of the amount provided via the feed, and for the YMW this varied from 89.0 to 95.6% (Table 4). The residues from the YMW also contained AFM1 when the larvae were fed 0.023, 0.084 mg/kg AFB1 or more, with excreted levels varying from 0.9 to 1.7% of the amount of AFB1 provided via the feed. This suggests that AFB1 was at least partially transformed in YMW to AFM1, which was subsequently excreted. Hence, the BSF larvae and YMW seem to metabolise AFB1 differently.

## 3. Discussion

This study evaluated the tolerance and accumulation of AFB1 in two insect species to determine whether they could be used to retain condemned mycotoxin contaminated crops in the food chain. The applied rearing conditions were successful in supporting the development of both insect species with high survival rates and normal growth. The mean body weight of the 10-day-old BSF larvae (152 mg) was similar to or slightly lower in older (14- to 21-day-old) larvae in other studies (171 mg in [23], 140–180 mg in [24]). For the YMW, the mean body weight was 119 mg, which is higher than the fifth and sixth instars reared on (lower quality) diets containing organic waste materials (78 mg in [20]) or industrial by-products (49–114 mg in [30]).

Both the BSF larvae and the YMW tolerated the tested dietary AFB1 levels, which suggests a higher tolerance than the corn earworm [17], and the first instar and 5-day-old larvae of the cabbage looper [18]. The extent of toxicity of AFB1 depends on the species-specific metabolism. AFB1 can be transformed by cytochrome P450 monooxygenases into the highly genotoxic AFB1-8,9-epoxide (AFBO) or into the less genotoxic metabolites AFM1, aflatoxin Q1, and aflatoxin P1 (AFP1) [31]. Furthermore, AFB1 can be reduced to aflatoxicol by NADPH-dependent aflatoxin B1 aldehyde reductases. The navel orangeworm metabolises AFB1 mainly to aflatoxicol along with AFM1 and aflatoxin B2a [32]. The absence of AFBO formation was suggested to underlie the high tolerance to AFB1 in this species [32]. The formation of aflatoxicol, AFM1, and AFB2a was also reported for a DDT-resistant strain of the fruit fly [33]. In the corn earworm, however, AFP1 was identified as the principal metabolite [34]. The BSF larvae and YMW seemed to metabolise AFB1 differently. The differences in pathways of AFB1 metabolism in BSF larvae and YMW underlying their tolerance to AFB1 warrant further study.

In case BSF larvae and YMW are used to retain condemned mycotoxin-contaminated crops in the food chain and act as high quality animal feed ingredients, it is essential that mycotoxins are not accumulated. The concentration of AFB1 in both insect species was very low. The BSF larvae fed the AFB1-containing feeds did not contain detectable levels of AFB1, i.e., below the detection limit of 0.10 µg/kg (Table 3). For the YMW, AFB1 was detected when they were provided with feed containing 0.023 mg/kg AFB1 or more. Concentrations ranged from 0.00011 to 0.00180 mg/kg dry matter. The YMW provided with poultry feed devoid of AFB1 and solvent for 2 days after being fed the feed with 0.415 mg/kg AFB1 contained less AFB1 than those harvested immediately (respectively 0.00041 ± 0.00004 and 0.00144 ± 0.00005 mg/kg dry matter, *p* < 0.001). This suggests that the gut contents contributed a relatively large part to the total AFB1 present in YMW, and shows that such a feeding strategy would further reduce AFB1 levels in YMW if required in practice. Based on the legal limit for feed material in the European Union (0.02 mg/kg, [10]), both the BSF larvae and YMW would have been allowed as animal feed ingredients. However, part of AFB1 might have been bound to proteins [29] and left undetected. Further information is therefore required on AFB1 metabolism, in order to ensure substrate detoxification. Finally, as mycotoxin-contaminated crops can contain multiple types of mycotoxins including the other aflatoxins, ochratoxins, trichothecenes, zearalenone, fumonisins, tremorgenic toxins, and ergot alkaloids [5], it would be of interest to study the tolerance and accumulation of individual and combinations of these mycotoxins in the selected insect species.

## 4. Conclusions

This study showed that the BSF and the YMW larvae are tolerant to AFB1 up to levels of 0.415 mg/kg of dry feed, which is approximately 20 times above the legal limit for feed material in the EU (0.02 mg/kg). Furthermore, larvae of the BSF did not contain AFB1, whereas the YMW contained AFB1 to levels that were approximately 10% of the legal limit. The metabolite AFM1 was not detected in the larvae but it was excreted by the YMW. Further studies are required to evaluate the presence of other AFB1 metabolites and the tolerance of these insect species to other mycotoxins. These findings of high AFB1 tolerance and apparent lack of accumulation warrant further studies in using BSF and YMW as a strategy to use condemned mycotoxin-contaminated crops in the food supply chain and to improve food security.

## 5. Materials and Methods

### 5.1. Feed Preparation

Standard poultry feed (AgruniekRijnvallei, Wageningen, The Netherlands) was sieved on a 2-mm screen and spiked with AFB1 (*Aspergillus flavus*, 99.6% purity, Sigma-Aldrich, Saint Louis, MO, USA) by an external laboratory (Ducares B.V., Utrecht, The Netherlands), which is ISO/IEC 17043 accredited as a proficiency testing organiser by the Dutch Accreditation Council for aflatoxins in animal feeds and animal feedstuffs (R009, RCS code 3). First, 2 mL of 0.25 g AFB1/L chloroform was diluted with 8 mL methanol, which was sprayed on and thoroughly mixed with 1000 g of poultry feed (i.e., 0.5 mg/kg of feed). The obtained spiked feed was used and mixed with AFB1-free feed to prepare the other treatment feeds with lower AFB1 concentrations of 0.25, 0.10, 0.05, 0.025, and 0.01 mg/kg of feed. Levels were chosen based on median and maximal AFB1 concentrations in feed ingredients from four Asian-Pacific regions (respectively 0.010 to 0.024 mg/kg and 0.275 to 0.457 mg/kg, *n* = 1291 samples), from three Europe and Mediterranean regions (respectively 0.012 to 0.019 mg/kg and 0.060 to 0.656 mg/kg, *n* = 1507) [35], and the EC maximum limits in feed materials (0.02 mg/kg relative to feed materials with 12% moisture) as defined in Directive 2002/32/EC [10]. One sample of pure poultry feed and one sample of poultry feed with solvent only (1 mL chloroform and 4 mL methanol in 400 g of poultry feed) were used as a control treatments. Feeds were stored at 5 °C prior to use.

### 5.2. Animal Procedures

All feeds were evaluated in triplicate by each insect species. For the BSF larvae, 18 g of the treatment feed was placed in a container (17.8 × 11.4 × 6.5 cm) and mixed with 36 mL of water. One hundred larvae, less than 24 h old from the colony of the Laboratory of Entomology (Wageningen University, Wageningen, The Netherlands) were added to the container. The container was then closed with a perforated lid, which allowed air exchange. These containers were arranged in crates in a tilting position and the crates were then stacked in a climate cabinet (27 ± 1 °C and 88 ± 1% relative humidity). The crates were rotated randomly three times per week to prevent effects of location in the climate cabinet. After 10 days, containers were weighed and larvae were collected, following which the boxes were weighed again. Boxes with residues (i.e., remaining feed, exuviae, excreta) were stored at −18 °C until further analyses. Collected larvae with attached residues were weighed, gently washed in a sieve with lukewarm water, dried using paper towels, and weighed again in order to determine the live weight of the larvae and the weight of the residue that adhered to their bodies. Larvae were then transferred to a new container containing 10 g poultry feed without solvent and AFB1 but with 20 mL tap water. After 2 days, larvae were collected, counted and cleaned according to the procedures described above, and stored at −18 °C until further analyses.

Fifty first instar YMW were placed in a plastic container (17.5 × 9.3 × 6.3 cm) with aeration slits in the sides and provided with 9 g of the particular treatment feeds. The containers were placed in stacked crates in a climate chamber at 28 °C and 70% relative humidity. These crates were rotated randomly three times per week to prevent effects of location. During the experiment, larvae were provided with increasing amounts of carrots (i.e., 0.3 to 1.75 g) three times per week. As soon as the first pupa was observed, the larvae and pupae were harvested per container and counted. The YMW and the residual material were then weighed, and stored at −18 °C until further analyses. To distinguish the amount of AFB1 in the gut from the larvae as a whole, three additional containers with 50 first instar YMW each were prepared and provided with the 0.5 mg AFB1/kg feed. These larvae were also removed from the container as soon as the first pupa was observed, and then placed in a new container with 4 g of poultry feed without solvent and AFB1. After 2 days, the larvae were harvested and processed as described above.

### 5.3. Sample Preparation and Aflatoxin Analyses

For feed, 2.5 g of sample material was weighed into a 250-mL Erlenmeyer flask. After the addition of 50 mL of chloroform (Actu-All Chemicals, Oss, The Netherlands) and 5 g of Celite (Boom, Meppel, The Netherlands), the mixture was shaken for 30 min on a rotating shaking apparatus. After filtration across a Whatman 595-1/2 folding filter (Brunschwig Chemie BV, Amsterdam, The Netherlands), 1.0 mL of extract was dried down (N_2_) and redissolved in 1.0 mL of methanol (Actu-All Chemicals, Oss, The Netherlands) and 9.0 mL of milliQ-water (Millipore, Amsterdam, the Netherlands).

For BSF larvae 2.5 g of frozen sample material and for the BSF residue 1.0 g of freeze-dried sample material was weighed into a 50 mL Greiner tube (Greiner Bio-One BV, Alphen aan de Rijn, The Netherlands). After addition of 12.5 mL of chloroform the mixture was homogenised for 1 min using an ultra-turrax. Celite (1.25 g) was added to the mixture and, after thorough shaking, the mixture was filtered across a Whatman 595-1/2 folding filter. Then, 2.0 mL from the organic extract was dried down (N_2_) and redissolved in 1.0 mL of methanol and 9.0 mL of milliQ-water. Larvae and residues of the YMW were oven-dried at 70 °C until a stable weight was reached. A subsample of 0.5 g was weighed into a 50-mL Greiner tube. After the addition of 12.5 mL of chloroform, the mixture was homogenised for 1 min using an ultra-turrax. Celite (1.25 g) was added to the mixture and, after thorough shaking, the mixture was filtered across a folding filter (Whatman 595-1/2). Then, 4.0 mL from the organic extract was dried down (N_2_) and redissolved in 1.0 mL of methanol and 9.0 mL of milliQ-water.

The total mixture of each of the sample extracts was then transferred to an Aflaprep (P07) immuno-affinity clean-up column (R-Biopharm, Darmstadt, Germany), which was washed twice with 5.0 mL of water. The aflatoxins were eluted with 2.5 mL of methanol and 7.5 mL of milliQ-water. The AFB1 and AFM1 analysis was performed by injecting 10 µL of the obtained extract in a Waters HPLC system (Waters Chromatography, Etten-Leur, The Netherlands), consisting of a Waters 2695 Alliance injector and Waters HPLC pump (flow 0.5 mL/min). The eluent consisted of a mixture of 600 mL milliQ-water, 255 mL methanol, 145 mL acetonitril (Actu-All Chemicals, Oss, The Netherlands), 114 mg KBr, and 61 µL concentrated HNO_3_ (VWR International BV, Amsterdam, The Netherlands). Chromatography was performed on a Waters Symmetry C18 (3.0 × 150 mm, 5 µm) HPLC column, and detection was performed after Kobra cell (1.5–2.0 V) oxidation, with a fluorescence detector (λ_ex_: 362 nm; λ_em_: 455 nm) (Jasco Benelux, De Meern, The Netherlands). The system was calibrated with certified reference solutions. Peak areas were used for quantification of the AFB1 and AFM1 contents, after recovery correction. The procedure was validated against certified reference material (AFB1) and spiked samples (AFB1 and AFM1). For the evaluation of the variation in AFB1 content of the feed, the AFB1 analysis of 0.5 and 0.25 mg/kg feeds was performed five independent times.

### 5.4. Calculations and Statistical Analyses

For each treatment, survival rate was calculated as living larvae at time of collection divided by the total larvae at the beginning of the trial (i.e., 100 for BSF and 50 for YMW) and multiplied by 100%. The larval body weight was calculated as the total weight divided by the number of larvae that survived. Results from quintuplicate AFB1 analyses of 0.25 and 0.5 mg/kg feeds were expressed as a coefficient of variation. For the treatments in which AFB1-containing feed was used, the AFB1 mass balance for feed, larvae, and residue (ng) was calculated as weight (g) × AFB1 content (mg/kg) × 1000. In case the AFB1 content in the larvae was below the detection limit of the analytical method (i.e., 0.0001 mg/kg), 0.0001 mg/kg was used in the calculation. The percentage of AFB1 lost was calculated as (AFB1_feed_ − AFB1_residue_ − AFB1_larvae_)/AFB1_feed_) × 100% with each parameter in ng. The percentage of AFM1 formed was calculated as (AFM1_residue_/AFB1_feed_) × 100% with each parameter in ng. The effect of solvent addition to the feed on larval body weight and survival, and the effect of providing YMW with AFB1-free feed for 2 days on their AFB1 contents were tested for significance using a one-way ANOVA by the GLM procedure (SAS Inst. Inc., Cary, NC, USA). Differences in larval weight and survival between the groups fed the poultry feed with only the solvent and those fed the feed with the solvent and AFB1 were also determined using one-way ANOVA. Data are expressed as means ± SD.

## Figures and Tables

**Table 1:** Formulated and analysed aflatoxin B1 contents (mg/kg) of spiked poultry feed.

Formulated	Analysed
0.01	0.013
0.025	0.023
0.05	0.049
0.10	0.084
0.25	0.204
0.50	0.415

**Table 2:** Mean body weight (mg) and survival (%) (±SD, *n* = 3 replicates) of black soldier fly (BSF) larvae and yellow mealworm (YMW) provided with feed without aflatoxin B1 and without or with solvent ^1^ for 10 days (BSF larvae) or approximately 40 days (YMW).

Insect	Parameter	Without Solvent	With Solvent	*p*-Value
BSF larvae ^2^	Body weight	152 ± 7	152 ± 14	0.972
	Survival	92.7 ± 4.6	95.5 ± 0.7	0.473
YMW	Body weight	119 ± 3	108 ± 8	0.088
	Survival	95.3 ± 2.3	96.7 ± 2.3	0.519

^1^ 0.25 mL chloroform and 1 mL methanol per 100 g poultry feed; ^2^ Data from one of three replicates fed poultry feed with the solvent was excluded from the analyses due to a miscount of larvae at the start of the trial.

**Table 3:** Mean (±SD, *n* = 3 replicates) aflatoxin B1 and aflatoxin M1 contents (µg/kg dry matter) in black soldier fly (BSF) larvae and yellow mealworms (YMW) provided with poultry feed spiked with varying levels of aflatoxin B1.

Insect	Compound	Aflatoxin B1 Content (mg/kg feed)					
		0.013	0.023	0.049	0.084	0.204	0.415
BSF larvae	Aflatoxin B1	<DL ^1^	<DL	<DL	<DL	<DL	<DL
	Aflatoxin M1	<DL	<DL	<DL	<DL	<DL	<DL
YMW	Aflatoxin B1	<DL	0.16 ± 0.06 ^2^	0.34 ± 0.14 ^2^	0.59 ± 0.23	1.29 ± 0.47	1.44 ± 0.05
	Aflatoxin M1	<DL	<DL	<DL	<DL	<DL	<DL

^1^ Aflatoxin B1 or M1 contents in all three samples were below detection limit (DL) of 0.10 µg/kg; ^2^ Based on *n* = 2, as one sample was below detection limit.

**Table 4:** Mean (±SD, *n* = 3 replicates) aflatoxin B1 (AFB1) mass balance (ng) and loss (%), and aflatoxin M1 (AFM1) (% of feed aflatoxin B1) in black soldier fly (BSF) larvae and yellow mealworms (YMW) provided with poultry feed spiked with varying levels of aflatoxin B1.

Insect	Parameter	Aflatoxin B1 Content (mg/kg feed)					
		0.013	0.023	0.049	0.084	0.204	0.415
BSF larvae	Feed	231 ± 0	419 ± 0	875 ± 0	1512 ± 0	3667 ± 0	7478 ± 1
	Insect	0 ± 0 ^1^	0 ± 0 ^1^	0 ± 0 ^1^	0 ± 0 ^1^	0 ± 0 ^1^	0 ± 0 ^1^
	Residue	10 ± 2	32 ± 17	47 ± 13	110 ± 56	275 ± 15 ^2^	1270 ± 201
	AFB1 lost	95.7 ± 0.9	92.3 ± 4.0	94.6 ± 1.4	92.7 ± 3.7	92.5 ± 0.4	83.0 ± 2.7
	AFM1 formed	<DL ^3^	<DL	<DL	<DL	<DL	<DL
YMW	Feed	115 ± 0	209 ± 0	437 ± 0	756 ± 0	1833 ± 0	3738 ± 0
	Insect	0 ± 0 ^1^	0 ± 0 ^1^	1 ± 0	1 ± 0	2 ± 1	2 ± 0
	Residue	5 ± 2	13 ± 2	35 ± 6	83 ± 6	139 ± 17	352 ± 114
	AFB1 lost	95.5 ± 1.4	93.7 ± 1.1	92.0 ± 1.4	88.8 ± 0.8	92.3 ± 1.0	90.5 ± 3.0
	AFM1 formed	<DL	1.7 ± 0.0 ^4^	<DL	0.9 ± 0.0 ^4^	0.9 ± 0.0	1.1 ± 0.0

^1^ For contents below the detection limit of the analytical method (see Table 3), the limit of 0.0001 mg/kg was used in the calculation; ^2^ Based on *n* = 2, as one sample as one sample extract did not pass the immuno-affinity clean-up column and was not analysed; ^3^ Aflatoxin B1 or M1 contents in all three samples were below detection limit (DL) of 0.10 µg/kg; ^4^ Based on *n* = 2, as one sample was below detection limit.
